# Involvement of Executive Functions in Idiom Comprehension: A Life-Span Perspective

**DOI:** 10.3390/brainsci14111076

**Published:** 2024-10-28

**Authors:** Agnès Lacroix, Nolwenn Troles, Mélissa Burgevin, Camille Le Bescond, Virginie Laval, Audrey Noël

**Affiliations:** 1 Laboratoire de Psychologie du Comportement, Cognition et Communication (EA 1285), University of Rennes 2, 35000 Rennes, France; nolwenn.troles@univ-rennes2.fr (N.T.); melissa.burgevin@univ-brest.fr (M.B.); camille.lb412@gmail.com (C.L.B.); audrey.noel@univ-rennes2.fr (A.N.); 2 Centre de Recherches en Cognition et Apprentissage (UMR CNRS 6234), University of Poitiers, 86000 Poitiers, France; virginie.laval@univ-poitiers.fr

**Keywords:** executive functions, pragmatic, comprehension, idiom, prefrontal cortex

## Abstract

Objectives—Our aim is to investigate the executive functions involved in idiom comprehension. The understanding of idioms has largely been explored from a developmental perspective. However, little is known about the cognitive processes involved. Recent studies highlight the contributions of working memory and inhibitory control in idiom processing. We investigated executive functions’ implication in idiom comprehension from a life-span perspective. Methods—The participants of this study were aged from 6 years to adulthood (*n* = 109 participants). An idiom comprehension task and executive tests were administered. Results and Conclusions—The results indicated that idiom comprehension improved across all the age groups tested. Moreover, the involvement of inhibition and cognitive flexibility processes was specific at different ages and particularly involved during adolescence.

## 1. Introduction

Idiom comprehension—Every day, we use forms of figurative language (metaphors, sarcasm, irony, idioms, proverbs) in our conversations. Figurative language allows us to communicate meaning that differs in various ways from what we would literally say [1]. An idiom is a stereotyped expression that is characterized by its conventional meaning and contextual conditions. Of course, an idiom can usually be interpreted literally, but when used in a specific context, it takes on a nonliteral meaning. For example, the literal interpretation of the French idiom Changer de disque is to “change the record”. In another context, however, it can be used figuratively to mean that the speaker wants to talk about something else (conventional meaning) [2,3]. Studies have shown that idiom comprehension begins in early childhood and gradually improves throughout the school-age years, adolescence, and well into adulthood [2]. Indeed, before being able to use idioms fully, children go through several stages. Thus, at the age of five, children start understanding idioms, and this skill continues to develop until the age of 12, while idiom explanation develops mainly between ages 6 and 11 and improves during adolescence [4,5,6,7]. However, previous studies focused on a single period of development (childhood or adolescence), and, to our knowledge, no study so far has tracked these abilities across childhood, adolescence, and adulthood. Several factors can influence the understanding of idiomatic expressions, such as the context in which communication happens, the familiarity between the people, and the transparency of the idiom. Familiarity is a measure of how frequently an expression occurs in a language [7,8]. Transparency is a measure of the similarity between an idiom’s literal and nonliteral meanings [8,9]. When the literal and nonliteral meanings are close, idioms are regarded as transparent, but when the meanings are unrelated, idioms are regarded as non-transparent. Idioms are easier to understand when they occur in linguistically supportive contexts, as opposed to unsupportive or absent contexts [5,10,11,12,13,14,15,16,17,18,19,20,21,22]. Context may facilitate the interpretation of figurative language by providing the necessary semantic information from which the listener can extract or infer the expression’s most appropriate meaning. Previous studies also showed that more familiar and transparent expressions are easier to understand than those that are less familiar and less transparent [3,5,7,8,12,13,14,19,23,24,25,26,27,28].

Involvement of neural substrates in idiom comprehension—While the left hemisphere (LH) has a crucial role in language processing, the right hemisphere (RH) also contributes to certain aspects of language. Based on studies in patients with RH brain damage, it was hypothesized that the RH plays a role in the comprehension of figurative language because these patients present more deficits [29,30,31,32,33]. Furthermore, Beeman’s coarse semantic coding theory [34,35] proposes that the LH is specialized in processing close semantic relationships, while the RH is specialized in processing close and coarse semantic relationships. According to this theory, the RH is involved in processing non-literal language, such as metaphors, because they tend to have more distant semantic relationships. Beeman’s coarse semantic coding theory predicts that figurativeness and familiarity will influence RH recruitment. Moreover, processing language that includes coarse semantic relationships may require working memory functions because of the complexity of the language, recruiting more high cognitive processes. Very few neuroimaging studies have been conducted on idioms, and they mostly highlight the involvement of the left or bilateral fronto-temporal networks, in particular, in the inferior frontal and middle temporal gyri [1,33,36,37,38,39,40,41,42]. Meta-analyses emphasize the role of the left inferior frontal gyrus in idiom processing [32,43,44,45]. Activating this region would allow for increased attention to processing idioms. Thus, the best meaning of the sentence would be selected according to the context and the stored meaning in memory, and the literal meaning would be inhibited [45]. The left temporal gyrus would thus reflect lexical–semantic retrieval, while the right temporal gyrus would play a role in certain phenomena of semantic integration [46]. In essence, neuroimaging studies found a more moderate contribution of RH in figurative and non-literal language processing. Nevertheless, it seems that both hemispheres play a key role in the interpretation of idiomatic expressions [1,39,47]. For example, less transparent idioms may involve several areas, including the RH superior parietal lobe and the LH cerebellum [32,48,49]. These extended activations, especially on the contralateral side, may be explained by an increased demand for cognitive resources [42]. Indeed, classical models of idiom processing [50] put forth that the literal meaning of sentences is first activated despite the context. If the literal sense is not compatible, then an alternative response is sought. This operation requires shifting, inhibiting, and updating processes that demand a large amount of cognitive resources [42,51].

Executive functions (EFs) and neural substrates—EFs organize, direct, and regulate cognitive activity, emotional responses, and behaviors during goal-directed actions. They comprise a set of interdependent processes [52], including inhibition, working memory, shifting (cognitive flexibility), and planning. Inhibition consists of curbing or suppressing dominant, prepotent, or automatic responses (cognitive or motor), in order to control attention or behavior and resist distraction or interference from irrelevant information in the environment [53]. It can relieve working memory by suppressing superfluous stimuli. Working memory is defined by Baddeley (2012) [54] as the ability to memorize and manipulate a limited amount of information over a short period of time and to update this information. Shifting is the ability to modify a mental pattern, adapt to a new task, or switch from one task to another [55]. Finally, planning is the ability to anticipate future events and design a strategy to achieve a set goal. It is widely acknowledged that the prefrontal cortex is responsible for a set of high-level cognitive abilities [56]. Several functional neuroimaging studies [57,58] highlight significant activation of the prefrontal cortex during the performance of EF tests. Anderson (2002) [59] highlighted that the integrity of the prefrontal cortex is a necessary condition for intact executive functioning. The first studies suggested the existence of the following dissociation in how working memory functions: LH brain regions are involved in verbal modality, whereas the processing of visuospatial information depends on the RH. However, studies suggest a more complex network and demonstrate similar frontoparietal activation patterns in both hemispheres across verbal and non-verbal manipulations [60,61], the role of right dorsolateral prefrontal cortex (PFC) for verbal [62], and spatial working memory updating [63]. Marti et al. (2024) [64] recently highlighted the role of the right lateral frontal cortex in verbal updating and the involvement of the right inferior parietal lobe and posterior temporal cortex in spatial updating. Wager and Smith (2003) [65] reported that these cerebral areas are related to manipulating information stored in working memory and, therefore. executive functions.

The PFC is also involved in social cognition [66]. Indeed, several studies have demonstrated a relationship between pragmatic abilities and the frontal lobe [67], specifically the prefrontal cortex [1]. Some researchers have also examined the link between EFs and social competence [68,69]. More precisely, researchers have established links between executive difficulties and the comprehension of idiomatic expressions in pathologies where the frontal regions are impaired (for schizophrenia, see [70,71]; for Alzheimer’s disease, see [72], for brain injury, see [31]; for Autism Spectrum Disorder, see [73]). Several neuroscience studies have suggested that the brain’s prefrontal and parietal structures have a longer maturation time frame that extends into adolescence and early adulthood [66]. From a developmental point of view, childhood and adolescence are characterized by changes in cognitive and behavioral processes in line with the maturation of brain areas involved in higher-order functions. Children quickly acquire social, cognitive, and emotional knowledge. It has been suggested that the first dozen years of life are especially spectacular in terms of developmental neural changes [74,75,76,77]. The expansion of white matter connectivity during development is considered a key event for EF maturation. Many EF processes increase during the transition between childhood and young adulthood because of enhanced connectivity with prefrontal cortical areas that mediate the regulation of cognition and emotion [78]. The connections of the prefrontal cortex with other cortical regions and subcortical brain areas become stronger because of increased white matter connectivity and are thought to promote EF [79,80]. Today, we can observe these changes through diffusion tensor imaging, an MRI-based neuroimaging technique [78,81,82]. An increase in myelin occurs alongside the development of enhanced projections to and from the prefrontal brain. Myelin is also a biomarker for the formation of cerebral networks that underlie complex functions such as cognitive flexibility, working memory, and social cognition [74,83].

From a developmental point of view, EFs are present early in life. They improve throughout childhood and become relatively mature in early adulthood [59,84]. According to Diamond’s (2013) [53] integrative model, executive components have differentiated, hierarchical, and interrelated developmental trajectories. The development of executive processes is, therefore, gradual [85]. The rudiments of EFs emerge within the first years of life [86] and continue to develop until adolescence and even early adulthood. The acquisition of certain functions such as inhibition (observed as early as 4 years and undergoes particular improvement between 5 and 8 years; [87]) and working memory (develops between 4 and 14 years; [88]) is thought to enable the development of other EFs, such as cognitive flexibility [87] and, at a later stage, complex planning (after age 12). Cognitive flexibility reaches adult-like levels at around 15 years of age. So, research indicates that executive processes are present early in life and enhanced throughout childhood. Nevertheless, although the developmental profile of EFs is still unclear, it is now widely accepted that executive functions are involved in school readiness, academic achievement, and social–emotional competence in children and adolescents [89,90,91,92,93].

Idiom comprehension and EFs: what are relationships?—Bosco et al. (2017) [94] investigated the relations between communicative–pragmatic disorders and executive functions in traumatic brain injury. Their results highlighted the role of working memory, cognitive flexibility, and planning in pragmatic difficulties. This was confirmed in Ouerchefani et al.’s (2024) study [95]. They showed that executive functions, especially inhibition, planning, and cognitive flexibility, were involved in pragmatic language comprehension. A recent exploratory study [96] looked at the cognitive variables involved in spoken idiom comprehension in an adult population. The following cognitive variables were explored: general speed processing, inhibitory control, working memory, cognitive flexibility, vocabulary, and fluid intelligence. Personality was also explored. The results confirmed the contributions of working memory, inhibitory control (in listening span tasks), and vocabulary in idiom processing. The study showed that processing idioms recruit several cognitive processes. Inhibition seemed to be particularly relevant. However, other studies are necessary to confirm the involvement of EFs in spoken idiom comprehension.

More recent research exists on reading idioms and EFs. For example, Arnon and Lavidor (2023) [97] investigated the role of cognitive control in processing ambiguous idioms in adults during a reading task. Their results show that reaction time was correlated with a cognitive control measure (stop signal task). More precisely, the better the inhibition ability, the faster the processing of ambiguities. In 2024, Carrol and Segaert [98] investigated the online processing of idioms and creative modifications regarding executive functioning (working memory and inhibitory control) among an adult population. Their study consisted of reading idioms, and a method of eye-tracking was used. The authors did not observe the effects of working memory and inhibitory control on idiom processing. Nevertheless, they observed an effect on processing speed.

To sum up, the classical models of idiom comprehension suggest the involvement of executive functioning in idiom processing, but modern techniques have not been used to observe the relationship objectively between these phenomena. Moreover, the developmental trajectory of EFs (linked to slow cerebral maturation) could account for the ongoing development of pragmatic competence into adulthood. That is why we believe it is crucial to explore the involvement of EFs in idiom comprehension across childhood and adolescent development. Therefore, in the present study, we investigated (1) the development of idiom comprehension and (2) the involvement of EFs in this comprehension. To our knowledge, this is the first investigation to take a developmental approach by studying idiom comprehension across a cross-sectional sample of children, adolescents, and adults.

## 2. Materials and Methods

### 2.1. Participants

A total of 109 individuals took part in this study, which was conducted from 2012 to 2015. They were divided into five age groups as follows: (1) a 6-year-old group composed of 16 children (7 boys and 9 girls; mean age = 6 years 7 months; SD = 7 months), (2) an 8-year-old group composed of 16 children (9 boys and 7 girls; M_age_ = 8 years 6 months; SD = 6 months), (3) an 11-year-old group composed of 18 children (8 boys and 10 girls; M_age_ = 11 years; SD = 10 months), (4) an adolescent group composed of 21 adolescents (7 boys and 14 girls; M_age_ = 16 years; SD = 11 months), and (5) an adult group composed of 38 adults (16 men and 22 women; M_age_ = 24 years; SD = 33 months). The participants were recruited in primary schools, secondary schools, and universities in Brittany (France). A blank copy of the informed consent form used in our study is in Supplementary Material.

### 2.2. Materials: Construction and Assessment

We collated a set of 50 idioms and ran a pilot study with two different samples of adults to gauge the familiarity and transparency of these idioms.

#### 2.2.1. Idiom Familiarity Task

In the idiom familiarity task, 15 undergraduate students were shown a list of the 50 idiomatic expressions. They were asked to assess the familiarity level of these idioms by estimating their frequency. This familiarity judgment task was similar to the one used by Nippold and Rudzinski (1993) [27]. Participants were each given a booklet containing the set of 50 idioms. They were then asked to indicate how often they had heard each expression before, on a 5-point scale (1 = never; 2 = once; 3 = a few times; 4 = several times; 5 = many times).

#### 2.2.2. Idiom Transparency Task

A different group of 15 undergraduate students were shown a list of 50 idiomatic expressions. The transparency judgment task was identical to the one used by Cain, Oakhill, and Lemmon (2005) [23]. For each expression, the figurative meaning was provided, and individual words and/or groups of words in the sentence were underlined (e.g., passer l’éponge [to wipe with a sponge], jouer avec le feu [to play with fire]). The participants’ task was to rate the extent to which the underlined components contributed to the figurative meaning on a 5-point scale, where higher scores indicated a greater contribution.

Based on these two series of ratings, we chose the 12 most familiar and transparent idioms and the 12 least familiar and transparent idioms. Table 1 lists our final selection of 24 idioms, together with their mean familiarity and transparency ratings.

### 2.3. Experimental Task

**Figure 1:**
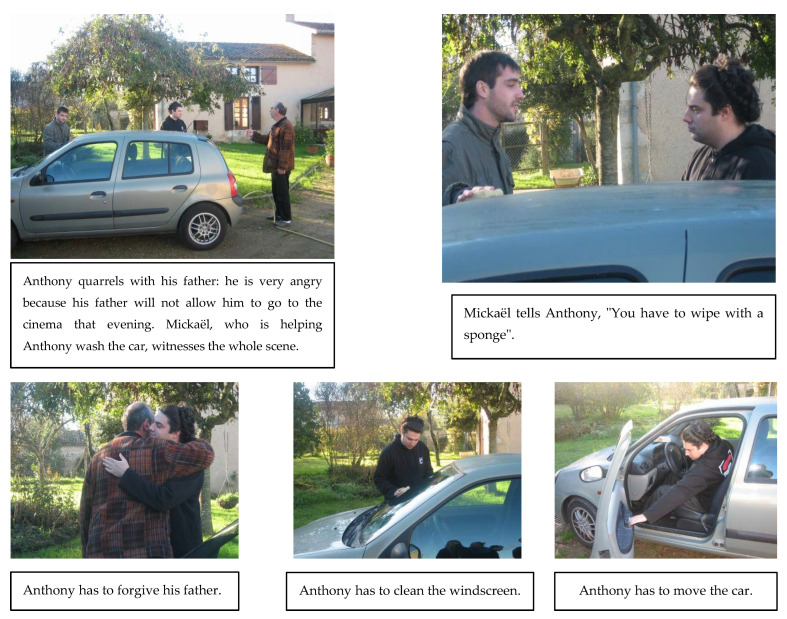
Screen capture of a story (idiomatic context) just before the participant responds.

We created one short story for each idiomatic expression. These 24 stories staged events experienced by two main characters (Anthony and Mickaël) in the course of their everyday lives. They varied in context (idiomatic vs. ambiguous), transparency level (transparent vs. non-transparent), and familiarity level (familiar vs. unfamiliar), and each was presented in three parts (see the example in Figure 1).

The first part supplied the context via a picture showing the two characters and a short oral description of the setting. We manipulated the context to be either idiomatic or ambiguous. An idiomatic context strongly induced an idiomatic interpretation, although the literal interpretation was still possible. In Figure 1, featuring the expression Il faut que tu passes l’éponge [You have to wipe the slate clean (with a sponge)], Anthony and Mickaël are washing a car, thus allowing for a literal interpretation. This was a key characteristic of our material, as the literal interpretation had to be appropriate even if it was not promoted by the story’s context. An ambiguous context did not sway the reader in favor of either an idiomatic or a literal interpretation. Instead, both interpretations were possible. In half the stories (12), the context was idiomatic, and in the other half (12), it was ambiguous. In the second part of the story, the idiomatic expression was produced, while the third part of the story constituted the story’s ending. For each story, we drew up three answers as follows: one answer represented the idiom’s idiomatic meaning (idiomatic answer); one was a paraphrased version of its literal meaning (literal answer); and the third one (situational answer) expressed a meaning that was plausible within the general context of the story.

### 2.4. Procedure

We propose the same protocol to all the participants. We assessed the participants’ comprehension of the idioms in a multiple-choice task in which they had to choose one of three interpretations of the idiom to finish the story. Multiple-choice tasks have been successfully used in the past to assess idiom comprehension [2,5,8,19,20]. This method makes fewer language production demands than an explanatory task; therefore, it is probably a more sensitive measure for comparing typical and atypical groups on idiom comprehension.

The task was administered on a laptop computer, and the entire experimental paradigm was computerized with E-Prime 2.0, which synchronized the images and sounds of the 24 stories (see Figure 1). Participants were tested individually in a quiet room where the experimenter seated them in front of the computer. The 24 stories, displayed one by one in random order, were preceded by two practice stories that helped the participants understand the task procedure. The first of two pictures appeared when the participant pressed the space bar on the keyboard. It was accompanied by its corresponding audio material describing the setting. Once the audio material for the first picture was played, the second picture was automatically displayed alongside the first one, showing the two characters closer together and Mickaël speaking with Anthony, again accompanied by its corresponding audio material.

Then, three pictures corresponding to the three possible choices (idiomatic, literal, situational) appeared in squares numbered 1–3 on the computer screen. Each picture was accompanied by its corresponding audio material. At this point, the participants had to choose one of these pictures by pressing the button on the keyboard that corresponded to the selected photo. The order of the three possible answers was counterbalanced across the 24 stories. Next, the participants had to explain or justify orally why they had chosen a particular ending.

### 2.5. Coding the Story Completion Answers (Comprehension)

The participants’ task was to finish each story by choosing one of three possible endings (idiomatic, literal, or situational). Idiomatic answers were the ones in which the audio material of the chosen picture expressed the idiomatic meaning of the expression. Given our study aims, we only analyzed the results for these answers. The number of answers therefore varied from one participant to another (maximum = 24).

### 2.6. Assessment of Executive Functions

Working memory was assessed by means of three span tasks [99,100]. In the verbal working memory task (assessing the phonological loop), the participants were asked to memorize a series of letters presented one at a time on a computer screen and immediately recall these letters in their order of presentation. Each letter was displayed for one second. Each series comprised three items of the same length, and the series increased in length from 3 to 11 letters. The test was stopped when the participants failed to correctly recall two items of the same length. The score corresponded to the length of the last correctly recalled item series. The same methodology was used for the spatial and multimodal span tasks. In the spatial span task (assessing the visuospatial sketchpad), the participants had to memorize the location of crosses that appeared randomly (one by one) in a 4 × 4 grid and then enter their response in a blank grid displayed on a computer screen. In the multimodal span task (assessing the episodic buffer), the participants had to memorize letters and their locations simultaneously in a 4 × 4 grid. These letters appeared on the screen one at a time, in one of the squares of the grid. The participants again had to enter their responses in a blank grid. So, the measures were verbal span, spatial span, and multimodal span.

Inhibition abilities were measured with the Stroop test [101,102] and the Hayling test [103,104]. In the Stroop test, the names of colors were printed in inks of different colors (e.g., the name red was printed in green ink). Instead of reading the name, participants had to say the color of the ink, inhibiting the automatic activity of reading. For this task, we calculated an interference coefficient using the formula developed by Golden (1978) [105]. In the Hayling test, the participants were asked to complete a sentence with a word that was not linked either to the sentence or to the expected word. The chosen word would have given the sentence an absurd meaning. In this test, we counted the number of mistakes (sentences completed with a word that was semantically linked to it).

To assess shifting processes, we used the Trail Making Test (TMT; [106]). The participants had to match as many alternating digits (1–13) and letters (A–L) as possible. For this task, we considered performance in terms of completion times.

Finally, we used the Wisconsin Card Sorting Test (WCST; [107]) to assess inhibition, shifting, and planning. Each participant was given a number of stimulus cards and told to match them, but not how to do so, although they were told whether a particular match was right or wrong. The participants had to find three possible rules (color, form, and number of items on the cards) and apply each one. We deemed the participants successful at finding one of the rules for the card matching if they matched them on the same criterion at least six times in a row and then found another criterion for matching them without being helped. In this task, we took perseverative errors into account.

Table 2 presents the results obtained for all the groups.

## 3. Results

Table 3 presents the results obtained for all the groups in each condition of the experimental task.

For the comprehension task, we ran an ANOVA on the proportion of idiomatic responses, with age group as the between-participants factor.

**Figure 2:**
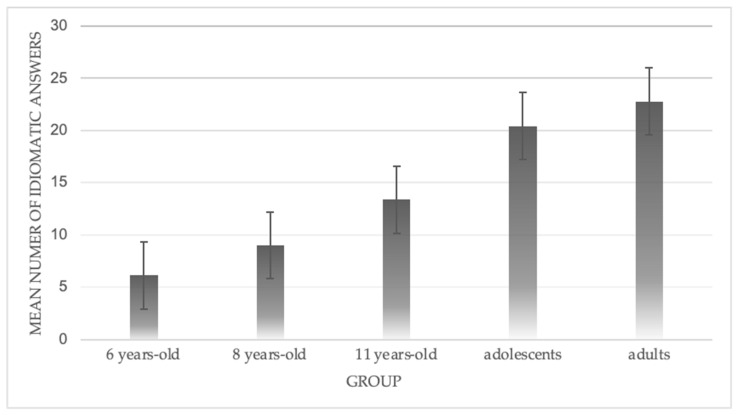
Mean number of idiomatic answers for each of the five groups.

We observed a significant group effect, *F*(4, 104) = 106, *p* < 0.001. We then ran Tukey post hoc tests, which indicated that the 6-year-olds gave fewer idiomatic answers than the 11-year-olds (*p* < 0.001), adolescents (*p* < 0.001), and adults (*p* < 0.001). The results also indicated that the 8-year-olds gave fewer idiomatic answers than the 11-year-olds (*p* < 0.01), adolescents (*p* < 0.001), and adults (*p* < 0.001). The 11-year-olds gave fewer idiomatic answers than the adolescents (*p* < 0.001) and adults (*p* < 0.001). Lastly, the adolescents did not differ from the adults. Figure 2 illustrates these results.

### 3.1. Effect of Context

We ran an ANOVA on the proportion of idiomatic responses, with age group as the between-participants factor and type of context (idiomatic vs. ambiguous) as the within-participants factor.

**Figure 3:**
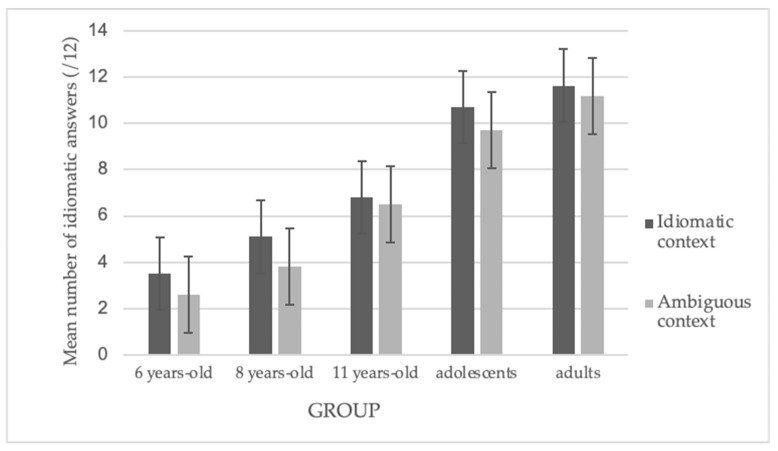
Mean number of idiomatic answers according to context.

We observed significant main effects of group, *F*(4, 104) = 109, *p* < 0.001, and context, *F*(1, 104) = 21.65, *p* < 0.001, indicating that idiom comprehension was better in an idiomatic context than in an ambiguous one. We did not observe a significant interaction effect. Figure 3 illustrates these results.

### 3.2. Effect of Familiarity

We ran an ANOVA on the proportion of idiomatic responses, with age group as the between-participants factor and degree of familiarity (familiar vs. unfamiliar) as the within-participants factor.

**Figure 4:**
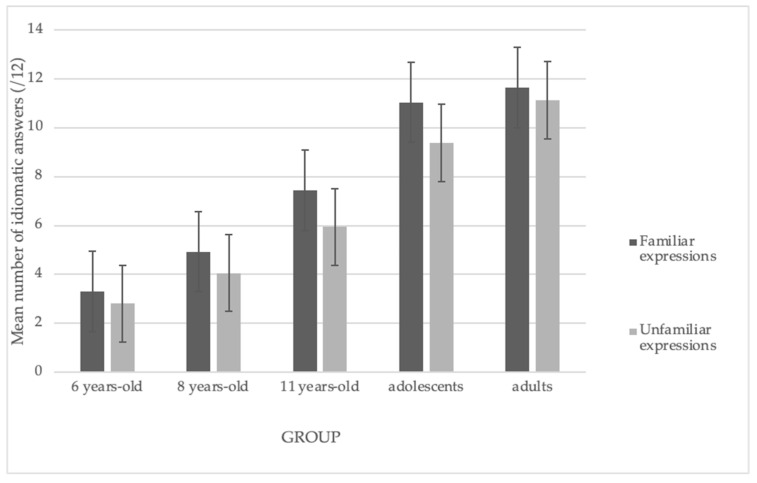
Mean number of idiomatic answers according to degree of familiarity.

We observed significant main effects of group, *F*(4, 104) = 108, *p* < 0.001, and familiarity, *F*(1, 104) = 29.62, *p* < 0.001, indicating that comprehension was better for familiar idioms than for unfamiliar ones. We did not observe a significant interaction effect. Figure 4 illustrates these results.

### 3.3. Effect of Transparency

We ran an ANOVA on the proportion of idiomatic responses, with age group as the between-participants factor and the degree of transparency (transparent vs. non-transparent) as the within-participants factor.

**Figure 5:**
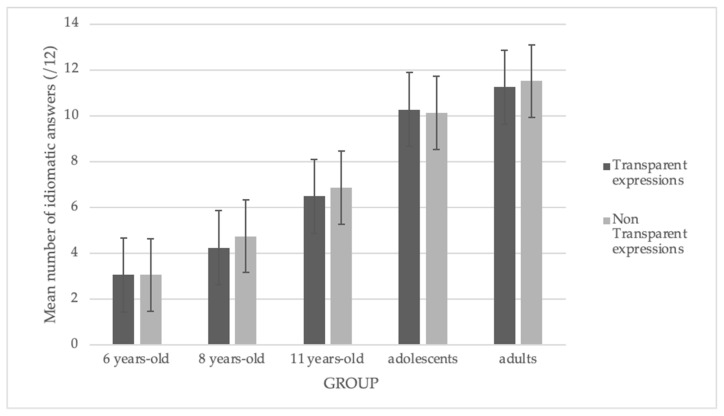
Mean number of idiomatic answers according to the degree of transparency.

We did not observe any significant effect of the transparency. We did not find a significant interaction effect. Figure 5 illustrates these results.

### 3.4. Relationship Between Idiom Comprehension, Working Memory, and Other EF Performances

We conducted bivariate correlations between the total number of idiomatic answers in all the conditions (total score on 24, score on 12 in idiomatic and ambiguous contexts, score on 12 for familial and unfamiliar expressions and for transparent and non-transparent idioms) and EF performance variables (working memory, inhibition, and flexibility). We considered the following levels of significance for *p*-values: (1) highly significant (*p* ≤ 0.001), (2) significant (*p* < 0.05), (3) marginally significant or trend-level significant (0.05 ≤ *p* ≤ 0.07), and (4) not significant.

For the 6-year-olds, we observed only one trend correlation between the number of idiomatic answers for non-transparent idioms and the Stroop interference score (*r* = 0.46, *p* = 0.07).

For the 8-year-olds, we observed a significant correlation between the number of idiomatic answers in an idiomatic context and the Stroop interference score (*r* = 0.52, *p* < 0.05). The better the inhibitor process, the better the idiom comprehension in an idiomatic context.

For the 11-year-olds, we did not observe any significant results.

For the teenagers, we observed significant correlations between the numbers of errors on the Hayling test and (a) the number of idiomatic answers in idiomatic context (*r* = −0.55, *p* < 0.05), (b) the number of idiomatic answers for familiar expressions (*r* = −0.50, *p* < 0.05), and (c) a trend negative correlation with the total number of idiomatic answers (*r* = −0.39, *p* = 0.07). We also observed significant negative correlations between the interference score on the Stroop test and the number of idiomatic answers for non-transparent expressions (*r* = −0.50, *p* = <0.01). We observed significant negative correlations between the number of perseverative errors on the WCST and (a) the total number of idiomatic answers (*r* = −0.53, *p* < 0.05), (b) the number of idiomatic answers in an idiomatic context (*r* = −0.56, *p* < 0.01), (c) the number of idiomatic answers for unfamiliar expressions (*r* = −0.48, *p* < 0.05), (d) the number of idiomatic answers for non-transparent expressions (*r* = −0.64, *p* < 0.01), and (e) a trend negative correlation with the idiomatic answers in ambiguous context (*r* = −0.40, *p* = 0.07). Moreover, we observed significant negative correlations between TMT completion time and (a) the number of idiomatic answers in ambiguous context (*r* = −0.44, *p* < 0.05), (b) the number of idiomatic answers for familiar expressions (*r* = −0.61, *p* < 0.01), (c) the number of idiomatic answers for transparent expressions (*r* = −0.59, *p* < 0.01), and (d) a trend negative correlation between TMT completion time and the number of idiomatic answers (*r* = −0.40, *p* = 0.07). Lastly, we observed significant negative correlations between TMT errors and (a) the number of idiomatic answers for familiar expressions (*r* = −0.44, *p* < 0.05) and (c) a trend negative correlation with the number of idiomatic answers for transparent expressions (*r* = −0.40, *p* = 0.06).

For adults, we only observed significant results for familiar expressions. There was a significant negative correlation between the number of idiomatic answers and the interference score on Stroop (*r* = −0.33, *p* < 0.05) and a trend significant correlation with TMT errors (*r* = −0.29, *p* = 0.07).

## 4. Discussion

The present study investigated the involvement of EFs in idiom comprehension. Further, the originality of this work lies in the adoption of a developmental perspective, as we studied these abilities from 6 years into adulthood.

Aligned with previous studies of idiom comprehension, our results showed that idiom comprehension continues to develop across childhood, adolescence, and early adulthood. They also showed that context facilitates idiom comprehension [5,10,11,12,13,17,19,21], as does familiarity [5,7,8,12,13,19,23,28]. Hamdan and Smadi (2021) [14] also found that context facilitated young children’s understanding of idioms and that older children were better able to understand idioms with or without context, highlighting their developing abilities. Interestingly, transparency had no effect on idiom comprehension.

Concerning the implication of EFs in idiom comprehension, our results indicated that the inhibitor process is particularly involved from the age of 6, especially for the more complex expressions (e.g., nontransparent idioms). Confronted with a complex language, children with good inhibition abilities will utilize them to access the idiomatic sense. In 8-year-olds, the inhibitor process is involved when idioms are presented in an idiomatic context. We can suppose that inhibition will be recruited to suppress the automatic response of literal interpretation. In 11-year-olds, no EF seems to be recruited. In contrast, during adolescence, inhibition and cognitive flexibility are particularly recruited in every condition. From there, in adulthood, inhibition and cognitive flexibility are solicited more specifically for familiar expressions. Our results suggest that working memory is not implicated in spoken idiom comprehension. Our results point to the conclusion that when we process an idiomatic expression, we begin by inhibiting its literal sense in order to switch to and access its nonliteral meaning. In terms of child development, we found that inhibition was significantly correlated with idiom comprehension at around 9 years, consistent with Anderson (2010) [108], indicating that this ability is well-mastered at this age. By adolescence, flexibility is fully functional, with fewer perseverative behaviors [108], and our results highlighted the involvement of this ability in idiom comprehension. Consistent with our results, recent studies comparing ASD and typical children have also found that executive functions, specifically mental flexibility, predicted idiom comprehension in typical children [73,109]. Our results lead us to believe that there is a specific developmental model for the ability to understand idioms. Indeed, not one or several unique executive functions contribute to idiom comprehension. Each executive function contributes at its own level, at different developmental times, and under different conditions (whether in relation to context or type of expression).

The development of idiom comprehension up to adulthood could be related to the slow maturation of the prefrontal cortex. Indeed, this cerebral region continues forming up to 30 years of age [110]. Furthermore, the prefrontal cortex involvement in idiom comprehension has already been pointed out in adults [1]. In parallel with prefrontal cortex maturation, EF development might explain the gradual improvement in idiom comprehension. From a developmental point of view, EFs evolve throughout childhood, finally reaching maturity in early adulthood [59]. This long-term developmental process can be attributed first to cerebral maturation, which is dramatic until approximately 12 years old, and second, to the lengthy maturation of the frontal cortex. White matter continues growing during childhood, particularly in the frontal cortex, which is involved in EF and language processing and therefore contributes to the development of EFs [110]. With age, children become increasingly capable of diverting their attention from salient stimuli that are not relevant to the situation to more relevant ones. As mentioned, a developmental timeline concerning the installation of each EF is still unclear. We would expect that it is associated with increased maturity of anterior, posterior, and subcortical brain regions. Moreover, Anderson et al. (1996, 2001) [111,112] highlighted developmental regressions between 11 and 13 years old, especially in self-regulation and strategic decision-making. This development has been associated with neurophysiological changes such as myelination and synaptogenesis in the prefrontal cortex [74,83].

Diamond’s (2013) [53] model postulates that each executive component undergoes a specific course of development. Accordingly, when we examined the correlations between idiom scores and EF measures, we observed changes in EF involvement. At certain ages, inhibition was particularly closely involved; during adolescence, a crucial period for EF development (due to frontal lobe maturation), cognitive flexibility also played a role in idiom comprehension. This result supports the idea that the efficiency of inhibition and flexibility processes could allow idiom comprehension to develop. Inhibition may be central in suppressing prepotent, salient interpretations in favor of those that are more appropriate in the context. Literal, irrelevant meanings may, in some circumstances, be more readily accessed than other, perhaps more appropriate, meanings, and the ability to inhibit them may, therefore, play an important role in nonliteral language understanding [113].

Our study shows that the development of idiom comprehension could depend not only on context but also on executive functioning reinforced by the maturation of the prefrontal cortex. Naturally, to confirm these hypotheses, other studies are needed, including studies with direct measures of brain activity such as optical imaging, EEG, or the like.

The question of how EFs are organized or structured in children remains of great interest from both fundamental and practical points of view. At the fundamental level, studying the structure of EFs contributes to mapping different human cognitive functions and their development. At the practical level, better knowledge of this structure would allow for developing assessment tests to be better adapted to children from different age brackets, which in turn would allow for better screening and diagnoses of different disorders, not to mention better measurement of the impact of different rehabilitative or remedial procedures. A better understanding of when and how executive functions play a role in language development, in general, is essential to propose pertinent cognitive remediations.

## 5. Limitations and Perspectives

The first limitation of this study is the small sample size of participants. The consequence could be a lack of consistency in our results. In the future, we may increase the number of participants to confirm our results, or we may conduct a longitudinal study from the age of 6 years to 18 years.

The second limitation concerns the material. We used a task with images, which could interfere with the idiomatic interpretation. The idiomatic context is likely affected by the visual literal interpretation. In the future, we could propose a task based on the same principles without visual support.

## Figures and Tables

**Table 1:** Mean familiarity and transparency ratings of the 24 idioms used.

French Idiom	Familiarity Score	Transparency Score
**Familiar and transparent idioms**		
Passer l’éponge Literal interpretation: Wipe with a sponge Idiomatic interpretation: Forgive someone	4.20	4.66
Ne dormir que d’un oeil Literal interpretation: Sleep with only one eye Idiomatic interpretation: Hardly be able to sleep	4.06	4.73
Jouer avec le feu Literal interpretation: Play with fire Idiomatic interpretation: Take a big risk	4.26	4.60
Gagner le gros lot Literal interpretation: Gain the best prize Idiomatic interpretation: Benefit suddenly from an exceptional stroke of luck	4.17	4.32
C’est un jeu d’enfant Literal interpretation: This is a game for children Idiomatic interpretation: It is easy	4.02	4.28
Jeter l’argent par les fenêtres Literal interpretation: Throw money out of the windows Idiomatic interpretation: Be very extravagant	4.21	4.06
**Familiar and non transparent idioms**		
Poser un lapin Literal interpretation: Place a rabbit [somewhere] Idiomatic interpretation: Stand someone up	4.01	1
Passer un savon à quelqu’un L Literal interpretation: Give soap to someone Idiomatic interpretation: Scold someone	4.40	1.53
Se lever du pied gauche Literal interpretation: Get up on the left foot Idiomatic interpretation: Be in a bad mood	4.23	1.25
Etre dans de beaux draps Literal interpretation: Be in nice sheets Idiomatic interpretation: Be in a right mess	4.53	1.33
A côté de la plaque Literal interpretation: Be next to a manhole cover Idiomatic interpretation: Make a mistake	4.07	1.05
Ne pas manquer d’air Literal interpretation: Not to lack air Idiomatic interpretation: Have a cheek	4.13	1.46
**Unfamiliar and transparent idioms**		
Pleurer dans le gilet de quelqu’un Literal interpretation: Cry into someone’s cardigan Idiomatic interpretation:Complain	1.73	4.20
Etre au bout du tunnel Literal interpretation: Be at the end of the tunnel Idiomatic interpretation: Be about to emerge from a difficult time	1.57	4.11
Donner sa chemise Literal interpretation: Give away one’s shirt Idiomatic interpretation: Be very generous	1.40	4.32
Avoir un bandeau sur les yeux Literal interpretation: Wear a blindfold Idiomatic interpretation: Be blind	1.46	4.60
Avoir les mains libres Literal interpretation: Have empty hands Idiomatic interpretation: Have the freedom to act	1.50	4.25
Accorder ses violons Literal interpretation: Tune one’s violins Idiomatic interpretation: Reach an agreement	1.66	4.33
**Unfamiliar and non transparent idioms**		
Bâtir des châteaux en Espagne Literal interpretation: Build castles in Spain Idiomatic interpretation: Form impractical projects	1.17	1.50
Avoir les dents longues Literal interpretation: Have long teeth Idiomatic interpretation: Be very ambitious	1.23	1.25
Monter à l’échelle Literal interpretation: Climb a ladder Idiomatic interpretation: Take people’s jokes seriously	1.46	1.13
Baigner dans l’huile Literal interpretation: Bathe in the oil Idiomatic interpretation: Everything is OK	1.40	1.46
Il y a à boire et manger Literal interpretation: There is something to drink and eat Idiomatic interpretation: There are some good and some bad things	1.53	1.40
Casser sa pipe Literal interpretation: Break one’s pipe Idiomatic interpretation: Die	1.43	1.05

Note. The familiarity score ranged from 1 (very unfamiliar) to 5 (very familiar). The transparency score ranged from 1 (less transparent) to 5 (highly transparent).

**Table 2:** Assessment of executive functions in the five age groups.

	6-Year-Olds	8-Year-Olds	11-Year-Olds	Adolescents	Adults
	M (SD)	M (SD)	M (SD)	M (SD)	M (SD)
Verbal span	3.44 (0.51)	4.19 (0.75)	4.33 (0.84)	5.19 (1.03)	5.35 (0.87)
Visuospatial span	3.56 (1.03)	3.75 (1)	4.44 (0.98)	5.23 (0.94)	5.05 (0.80)
Multimodal span	2.75 (0.68)	3.50 (0.63)	3.94 (0.80)	4.76 (0.70)	4.63 (0.78)
Hayling test Number of errors	9.25 (2.86)	10.44 (3.26)	9.38 (2.11)	7.14 (3.70)	5.36 (2.95)
Stroop test Interference score	3.14 (7.30)	−0.5 (6.36)	−1.21 (4.95)	6.28 (8.14)	9.5 (12.02)
Wisconsin test Number of perseverative errors	5.75 (4.28)	5.81 (5.34)	3.89 (5.89)	2.71 (4.02)	0.60 (0.75)
Trail Making Test Duration Part B (ms)	306.13 (186.39)	118.38 (42.67)	90.88 (30.59)	60.09 (20.74)	57.23 (20.35)
Number of errors	1.88 (1.58)	0.38 (0.71)	0.33 (0.76)	0.38 (0.97	0.05 (0.22)

**Table 3:** Mean number of correct responses obtained in the five age groups (standard deviation is in parentheses).

Group	Total Score of Idioms (Max = 24)	Idiomatic Context (Max = 12)	Neutral Context (Max = 12)	Familiar Expressions (Max = 12)	Unfamiliar Expressions (Max = 12)	Transparent Expressions (Max = 12)	Non-Transparent Expressions (Max = 12)
6-year-olds	6.13 (3.93)	3.50 (2.58)	2.63 (1.96)	3.31 (2.65)	2.81 (1.76)	3.06 (2.32)	3.06 (2.21)
8-year-olds	9.00 (4.83)	5.19 (2.90)	3.81 (2.54)	4.94 (3.13)	4.06 (2.52)	4.25 (2.82)	4.75 (2.74)
11-year-olds	13.4 (4.74)	6.89 (2.93)	6.50 (2.09)	7.44 (2.83)	5.94 (2.24)	6.50 (2.60)	6.89 (2.52)
Adolescents	20.4 (2.09)	10.7 (1.01)	9.71 (1.35)	11 (0.97)	9.38 (1.60)	10.3 (1.42)	10.1 (1.15)
Adults	22.8 (1.30)	11.6 (0.67)	11.2 (1.01)	11.7 (0.53)	11.1 (1.02)	11.3 (0.92)	11.5 (0.64)

## Data Availability

The original contributions presented in the study are included in the article/Supplementary Material, further inquiries can be directed to the corresponding author.

## Supplementary Materials

The following supporting information can be downloaded at: https://www.mdpi.com/article/10.3390/brainsci14111076/s1.
