# Supplementary material for: Involvement of Executive Functions in Idiom Comprehension: A Life-Span Perspective
Source: Brain Sci. 2024 Oct 28;14(11):1076. doi: 10.3390/brainsci14111076 (PMC11592149; doi:10.3390/brainsci14111076)
Supplement: Supplementary file 1 [file brainsci-14-01076-s001.zip › brainsci-3229681-supplementary.pdf]

UNIVERSITE RENNES 2 HAUTE BRETAGNE

UFR **SCIENCES HUMAINES**

Département de Psychologie

Thématique de la recherche : La compréhension des expressions idiomatiques.

Détails du projet :

Dans toute situation de la vie quotidienne, nous sommes confrontés à une forme de langage bien particulière : le langage non littéral. Cette forme de langage repose sur une constante : il existe un décalage entre ce qui est dit et ce que l'on veut vraiment dire. Il en existe plusieurs types parmi lesquels l'ironie, le sarcasme, l'humour, et les expressions idiomatiques.

Les expressions idiomatiques ont fait l'objet de beaucoup de recherches notamment chez l'enfant afin de caractériser le développement de leur compréhension. Ces recherches ont globalement montré que la compréhension des expressions idiomatiques dépend du contexte et n'est acquise que vers l'âge de 9 ans.

Cependant, il est clairement souligné que certaines fonctions cognitives entrent en jeu : la mémoire de travail ou encore les fonctions exécutives. Le concept de **mémoire de travail** fait référence à un système dynamique, qui intègre non seulement la rétention de l'information, mais aussi sa transformation comme conséquence de l'activité cognitive. Dans l'acquisition et l'exécution des capacités cognitives de base, la mémoire de travail est souvent considérée comme jouant un rôle central. Elle est associée au développement d'activités cognitives complexes, comme la compréhension du langage, la lecture, la production écrite, le calcul ou le raisonnement. Par exemple, la mémoire de travail est impliquée dans le calcul mental : l'individu doit mémoriser les informations (en sélectionnant les plus pertinentes) et effectuer une opération sur celles-ci. Plus la mémoire de travail est efficiente, plus cette opération est aisée. Les **fonctions exécutives** recouvrent tout un ensemble de processus dont la fonction principale est de faciliter l'adaptation du sujet aux exigences et fluctuations soudaines de l'environnement et, en particulier, aux situations nouvelles. Elles constituent de véritables fonctions régulatrices du comportement. Parmi les fonctions exécutives, nous pouvons distinguer l'inhibition qui consiste à empêcher le traitement d'une information de façon automatique afin d'autoriser un autre traitement. Par exemple, lors d'une tâche de lecture, il peut être demandé de ne lire que les mots commençant par la lettre L (il faut donc empêcher l'automatisme de la lecture). Enfin, une autre capacité nous intéresse : la flexibilité mentale. Il s'agit ici d'être en mesure de passer d'une consigne à une autre. Par exemple, en situation scolaire, l'enfant doit être capable de passer d'un exercice à un autre alors que les consignes et le travail demandé est différent.

Notre étude a pour but de déterminer le rôle de la mémoire de travail et des fonctions exécutives dans la compréhension des expressions idiomatiques par les adolescents.

Pour étudier cela, nous proposons différentes tâches :

1- une estimation de la mémoire de travail sous la forme de 3 exercices (10 minutes)
2- une estimation des fonctions exécutives : inhibition et flexibilité mentale (15 minutes)
3- l'étude des expressions idiomatiques à travers un matériel novateur :

Des scénarios mettant des personnages sont présentés. Ces scénarios sont tous construits de la même façon : présentation du contexte, production de l'énoncé et la fin du scénario qui doit être complétée par les participants (3 réponses sont possibles).

Ainsi, nous déterminerons les capacités de compréhension des expressions idiomatiques et les capacités des adolescents à prendre en compte le contexte pour s'adapter au mieux à leur environnement social.

Ce type de recherche est particulièrement important pour accroître nos connaissances sur le développement du langage chez l'adolescent et par la suite, mieux comprendre les troubles observés chez des adolescents présentant des pathologies et améliorer leur prise en charge.

Document à faire remplir aux parents.

Je soussigné(e), ...,
autorise XX, à rencontrer mon enfant ....

Date

Signature
